# Exposure to high thermal conditions for a long time induces apoptosis and decreases total RNA concentration in peripheral blood mononuclear cells among Indian Zebu–Jersey crossbreds

**DOI:** 10.14202/vetworld.2022.2192-2201

**Published:** 2022-09-15

**Authors:** Gbolabo Olaitan Onasanya, George M. Msalya, Aranganoor K. Thiruvenkadan, Nagarajan Murali, Ramasamy Saravanan, Angamuthu Raja, Moses Okpeku, Mani Jeyakumar, Christian O. Ikeobi

**Affiliations:** 1 Department of Animal Science, Federal University Dutse, Dutse, Nigeria; 2 Department of Animal Genetics and Breeding, Veterinary College and Research Institute, Tamil Nadu Veterinary and Animal Sciences University, Chennai, Tamil Nadu, India; 3 Department of Animal, Aquaculture and Range Sciences, Sokoine University of Agriculture, Morogoro, Tanzania; 4 Department of Veterinary Microbiology, Veterinary College and Research Institute, Tamil Nadu Veterinary and Animal Sciences University, Chennai, Tamil Nadu, India; 5 Discipline of Genetics, School of Life Sciences, College of Agriculture, Engineering and Sciences, University of KwaZulu-Natal (Westville Campus), Durban, South Africa; 6 Department of Animal Breeding and Genetics, Federal University of Agriculture, Abeokuta, Nigeria

**Keywords:** bovine, cell survival, climate change, peripheral blood mononuclear cells, RNA synthesis, thermal stress

## Abstract

**Background and Aim:**

Global warming has grave consequences on livestock production systems and profound negative effects on animal production. This study aimed to carry out an *in vitro* thermal stress stimulation (TSS) of bovine peripheral blood mononuclear cells (PBMCs) using different thermal assault conditions (TACs), including normal to extreme temperatures and varying durations of thermal exposure (DTE) to understand how PBMCs of Indian Zebu–Jersey crossbreds respond to various levels and durations of heat shock.

**Materials and Methods:**

Ten milliliters of blood were collected from 70 Indian Zebu–Jersey crossbreds under aseptic conditions and were sampled for isolating PBMCs. Peripheral blood mononuclear cells were divided into seven groups, each comprising 10 PBMC samples isolated from 10 different animals. Aliquots of 500 μL of PBMCs were stressed by exposure to different TACs (37, 40, and 45°C) for DTEs of 3 or 6 h. Subsequently, the cells were harvested. The control unstressed samples (500 μL aliquots of PBMCs) were exposed to no TAC (0°C) and zero DTE (0 h). Total RNA from all the treatment groups of PBMCs were isolated and quantitated.

**Results:**

We found a very strong association between TACs and RNA levels. In addition, PBMCs viability was negatively affected by heat shock. This led to an exponential reduction in PBMC count as TACs toughened. Only 3.59 × 10^5^ ± 0.34 cells/mL were viable after exposure to 45°C for a 6 h DTE. This cell viability was lower than that measured in controls subjected to no stress and zero time DTE (2.56 × 10^7^ ± 0.22 cells/mL). We also observed a reduction in the concentration of RNA isolated from thermally stressed PBMCs.

**Conclusion:**

*In vitro* TSS of PBMCs provided biological information on the response of cellular systems to heat shock after exposure to TACs. This will help to mitigate and manage the effects of thermal stress in bovine species. The association between the reduction in PBMC count after *in vitro* TSS and the expression of heat shock protein 70 gene will be investigated in the future to further understand how Indian Zebu–Jersey crossbreds respond to *in vitro* thermal conditions. This will be used to determine the *in vivo* response of Indian Jersey crossbreds to different environmental thermal conditions and will further enable the *in vivo* understanding of thermotolerance potentials of bovine species for better adaptation, survival, and production performance.

## Introduction

Over the past few decades, global warming had grave consequences on livestock production systems and profound negative effects on animal production [1]. In the face of the existential threat posed by climate change, the survival of humans and animals has been challenged by environmental thermal conditions, leading to various consequences on optimal performance, production, and food security [2]. The impact of thermal stress (TS) on cattle can be overwhelming because these animals can succumb to hyperthermia if they fail to abate the impact of thermal assaults, which leads to reduced feed intake, low milk production levels, stunted growth, poor health, decreased activity, and poor performance [3]. Moreover, under such circumstances or harsh environmental conditions in the tropics, livestock animals are forced to increase their respiratory rate and peripheral blood flow, leading to negative effects on physiological and production performance, including poor milk quality. On the other hand, increases in environmental temperatures and relative humidity affect the ability of cattle and other animals to maintain homeostasis, a situation which forces them to actively maintain an internal body temperature (IBT) required for survival and production [4]. Homeothermy in animals is the thermoregulation of the IBT regardless of environmental thermal challenges, and TS is achieved when the animals’ body temperature (BT) is elevated beyond normal physiological levels. The situation causes increased management costs, affects food security, and negatively impacts income generation, thus resulting in economic loss [5].

Previous studies [6–9] reported the effects of variations in thermal assault conditions (TACs) and heat shock on cellular integrity, proliferation, and viability as well as on RNA concentration, thus predisposing animals to vulnerable opportunistic infections resulting from immune response breakdown and decline in production performance and reproduction. RNA as a nucleic acid is heat labile and becomes very unstable and very unstable under harsh environmental conditions, particularly thermal conditions. Furthermore, harsh environmental TACs were reported to cause degradation of RNA nucleotides, and subsequent mutational damages to nucleic acid structure impairing nucleic acid synthesis and functions. Additional reports showed that the synthesis and proliferation of RNA nucleotides are largely influenced by variations in temperature. For example, a moderate *in vitro* temperature of 37°C mimics the BT of mammals and enhances the synthesis and proliferation of RNA and viability of cells [8, 10]. Indeed, there is a need to mitigate the consequences of harsh environmental circumstances to enable livestock to perform better in terms of production and reproduction capabilities [3]. A first focus might be on improving traits such as fertility, conception rates, feed intake, growth, milk production, meat production, and animal health [6]. To achieve this, further biological information including various genomic regions and cellular systems enabling a wide range of farm animals to withstand TS must be obtained [2].

To contribute to the knowledge regarding cell stress in animals, we carried out an *in vitr*o TS stimulation (TSS) of bovine peripheral blood mononuclear cells (PBMCs) under different TACs and varying durations of thermal exposure (DTEs). The present preliminary *in vitr*o study focused on the impact of differential TACs on the cellular systems of Indian Zebu-Jersey crossbreds, and to investigate the effects of TSS on RNA degradation and concentration. Circulating PBMCs include leukocytes and can be used as a cellular model to understand how livestock animals respond to varying TACs, especially in a tropical environment [12]. *In vitro* TSS allows to investigate how cellular systems respond to heat shock and TACs typical of a tropical environment.

## Materials and Methods

### Ethical approval

The study was approved (approval no. (TWAS-DBT/FR number:3240313769/PDF/2021) by the post-doctoral research supervisory team led by Prof A. K. Thiruvenkadan (Host Supervisor) and this met laid down ethics for carrying our animals’ research by the institution.

### Study period and location

This study was conducted from October 2021 to March 2022 at Livestock Farm Complex, a teaching and research farm of Veterinary College and Research Institute (VCRI), Namakkal, Tamil Nadu Veterinary and Animal Sciences University, Chennai, India. The laboratory activities were performed at Post Graduate Laboratory at Department of Animal Genetics and Breeding, VCRI, Namakkal, Tamil Nadu Veterinary and Animal Sciences University, Chennai, India.

### Description of the study area and meteorological data

Longitude and latitude of the study area is 11°09’46.7”N 78°09’34.9”E. Sampling duration was between the hours of 1 pm and 2 pm when the average ambient temperature was 39°C. The study area experiences regular annual rainfall that ranges from around 640 mm to 880 mm, with a mean precipitation of 776 mm. The climate is typically semi-arid and tropical. It is hot all year round and oppressively gloomy during the wet season and humid and partially cloudy during the dry season. The average annual temperature ranges between 30°C and 43°C.

The average wind speed of the study area ranges between 4 and 6 km/h, while ambient humidity ranges between 65% and 90%. The wind speed seldomly increases to 15 km/h, and the annual wind gust ranges between 40 and 44 km/h. These meteorological data were obtained from the Agromet Field Unit of VCRI, Namakkal, Tamil Nadu Veterinary and Animal Sciences University, Chennai, India.

### Experimental animals and collection of biological samples

**Figure-1:**
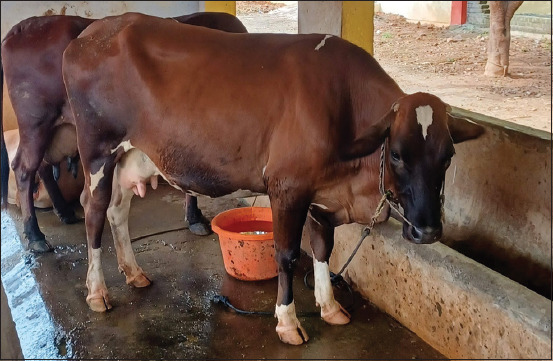
India Zebu–Jersey crossbred cattle.

Blood samples (10 mL per animal) were collected aseptically from 70 Indian Zebu–Jersey crossbreds aged 4–6 years (Figure-1). The animals were raised under an intensive system and given green forages as a basal diet and concentrates as a supplement. Clean water was provided *ad libitum*. After blood collection, the samples were immediately transported within a distance of 100 m (approximately 5 min) to the laboratory at the Department of Animal Genetics and Breeding, VCRI where PBMCs were isolated

### Isolation of PBMCs

Animal blood samples (10 mL) were homogenized and allowed to mix thoroughly. Homogenized blood was then poured into previously prepared 10 mL phosphate-buffered saline (PBS) (HiMedia, Laboratories, Mumbai, India) in a clean conical tube at an equal V/V ratio. This was followed by a gentle homogenization and thorough mixing by pipetting up and down. Subsequently, the blood-PBS mixture was carefully added to 3 mL of Histopaque^®^-1077 (Sigma-Aldrich Co. LLC, Darmstadt, Germany) in a new 50 mL conical tube and centrifuged at 400× g for 20 min in a REMI R-4C laboratory centrifuge (Goregaon East, Mumbai - 400 062, India).

**Figure-2:**
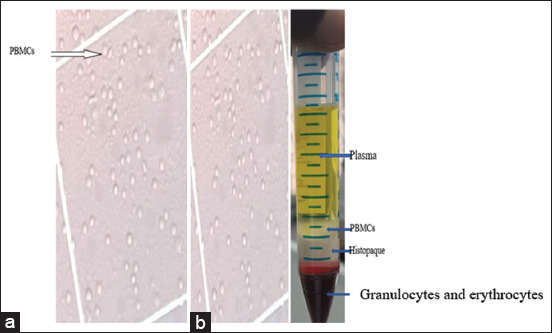
(a) Microscopic detection of peripheral blood mononuclear cells using hemocytometer viewed under microscope, (b) Fractional separation of bloods sample showing peripheral blood mononuclear cells and other fractional layers.

In fractional separation of fresh whole blood using Histopaque^®^-1077 (Sigma-Aldrich Co. LLC); four distinct layers were visibly obtained, including upper yellowish plasma layer, the milky PBMCs layer which was directly beneath the plasma layer, the histopaque layer, and the bottom layer containing erythrocytes-granulocytes (Figure-2).

After centrifugation, PBMCs were recovered with as little plasma as possible (and less Histopaque^®^-1077) and transferred into a clean new 15 mL conical tube. The PBMC suspension was then washed by adding 10 mL PBS and homogenizing thoroughly by pipetting up and down before centrifugation at 100× *g* for 10 min. Afterward, the supernatant was discarded to recover the PBMC pellet. This was followed by the flicking of the conical tube until the PBMC pellets were completely resuspended in the remaining PBS solution. Subsequently, 10 mL PBS solution was added to the pellet and mixed thoroughly by up-and-down pipetting. The mixture was centrifuged at 100× *g* for 10 min, and the supernatant was discarded.

The recovered PBMCs pellet were subjected to the procedure (washing with PBS, mixing, and centrifuging at 100× g for 10 min was repeated for third time Finally, 1 mL of a solution made of 900 μL basic media (RPMI-1640): 900 μL (HiMedia, Laboratories) with fetal bovine serum: 100 μL (HiMedia, Laboratories) was added to the PBMCs. The cells were gently resuspended by up-and-down pipetting. The number and viability of the isolated PBMCs were measured using the trypan blue dye exclusion method and the PBMCs were immediately subjected to TSS.

### Procedures for generating different TACs

Seventy animals divided into seven groups (10 animals per group) were examined in the study. Seventy aliquots of PBMCs, including stressed and unstressed cells, were obtained from blood samples of the 70 Indian Zebu–Jersey crossbred cattle breeds. The cells were exposed to four different TACs (0, 37, 40, and 45°C) for 3 h and 6 h, except for 0°C TAC to which they were exposed for 0 h. The concentration of viable cells was estimated to be within the range of 7.04 × 10^6^–2.56 × 10^7^ cells/mL before the TSS procedure. Each aliquot of 500 μL contained about 1 × 10^6^ PBMCs/mL. Initially, all PBMC aliquots were incubated in a nutrient medium (RPMI 1640) at 37°C for 30 min in a 5% CO_2_ incubator (Cole-Parmer Binder C170UL-120V-R CO_2_ Incubator, Mumbai, India) for stabilization. Then, the control sample labeled unstressed was immediately harvested after completion of 30 min of initial stabilization of both stressed and unstressed samples in nutrient media at 37°C in 5% CO_2_ incubator.

### Assessment of the cell number and viability

After PBMCs isolation, PBMCs number and viability were estimated using Trypan blue dye exclusion method. Trypan exclusion dye method involved the staining of PBMCs with trypan blue dye such that the viable PBMCs were not stained with Trypan blue dye, whereas the dead cells were stained and viewed on hemocytometer (Microyn Improved Neubauer Hemocytometer, Hunt Valley, Maryland, USA) under a microscope (CELESTRON Labs CB2000C Compound Microscope, Celestron, LLC., California, USA). The concentration of viable cells was estimated to be within the range of 7.04 × 10^6^ –2.56 × 10^7^ cells/mL before TSS.

### Thermal stress stimulation procedures

**Figure-3:**
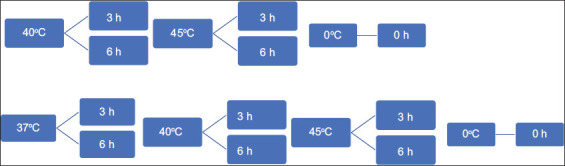
Experimental design showing differential thermal assault conditions and varying durations thermal exposure for stressed and unstressed peripheral blood mononuclear cells of Jersey crossbred cattle breed.

Isolated PBMCs were divided into two groups: One group was subjected to TS and another one that was not stressed. Aliquots of (500 μL) PBMCs at about 1 × 10^6^ PBMCs/mL were initially incubated in a nutrient medium (RPMI 1640) at 37°C for 30 min in a 5% CO_2_ incubator for stabilization. We carried out an *in vitro* TSS of PBMCs under differential TACs and DTEs. As shown in Figure-3, the PBMCs aliquots (500 μL) were labeled and subjected to four different TACs in a circulating REMI RSB-12 water bath: Four treatment groups (no TS: Control, 37°C: Normal temperature, 40°C: Moderate heat, and 45°C: Extreme temperature) and two DTEs (3 and 6 h).

After completion of TSS, the stressed PBMCs were allowed to recover at 37°C for 30 min in a 5% CO_2_ incubator before being harvested using trypsinization. In contrast, unstressed control samples (500 μL) were exposed to no TAC (0°C) and zero DET (0 h) and were harvested immediately after 30 min of stabilization. Stressed and unstressed PBMCs were immediately used for total RNA isolation. Total RNA was quantitated using Thermo-Scientific-Nano Drop 2000 spectrophotometer (Shimadzu co-operation, Kyoto, Japan).

### Estimation of viable peripheral blood mononuclear cells and concentration of viable PBMCs

The PBMCs were estimated, and viability was checked using various parameters shown in Formulae-1–4.

% Viability of PBMCs = Total viable PBMCs/Total number of PBMCs (Viable + Dead) × 100 (1)

Average number of viable PBMCs per square = Total number of viable PBMCs in 4 Squares/4 (2)

Dilution factor = Total volume (Volume of PBMCs + Volume of trypan blue dye/Volume of PBMCs (3)

Concentration of Viable PBMCs/Square = Average number of viable PBMCs X Dilution factor × 10^4^ (4)

For this study, two dilution factors were used.

### RNA purification

Total RNA was immediately extracted from stressed and unstressed PBMCs using a Trizol-based assay. A suspension of 250 μL PBMCs was added to 750 μL RNAiso plus (TaKaRa Bio Inc., Shiga, Japan) into a 1500 μL Eppendorf tube, thoroughly mixed, and incubated at room temperature (20-25°C) for 15 min. Then, 200 μL of chloroform (HiMedia, Laboratories) was added. The mixture was briefly vortexed for 5 s using a REMI Cyclomixer (Goregaon East) and subsequently incubated at 20-25°C for 15 min. After incubation, the samples were centrifuged at 1200× g for 15 min at 4°C. Afterward, the top aqueous supernatant was recovered and transferred into a new sterilized 1500 μL Eppendorf tube.

A volume of cold isopropanol (HiMedia, Laboratories, Mumbai, India) was added to the recovered supernatant in a V/V ratio, thoroughl**y** mixed by pipetting up and down, incubated a**t** 20-25°C for 10 min, and centrifuged at 1200× *g* for 10 min at 4°C. After centrifugation, the supernatant was discarded. A volume of cold 75% ethanol (HiMedia, Laboratories) equivalent to that of the discarded supernatant was added to the remaining isopropanol in the Eppendorf tube in a V/V ratio. The samples were thoroughly mixed by pipetting up and down and centrifuged at 7500× g for 5 min at 4°C. After centrifugation, the supernatant was discarded. The RNA pellet was recovered and left to dry at 20-25°C for 5 min.

Finally, 100 μL of RNase-free molecular grade water was added to dissolve the RNA pellet by thorough up-and-down pipetting. The RNA concentration and purity were measured using a Thermo-Scientific-Nano Drop 2000 spectrophotometer (Shimadzu Corporation, Kyoto 604-8511, Japan). The RNAs with purity ratios of 1.7–2.1 were used for further analyses. After the quantity and quality checks, isolated RNA was immediately used for post-TSS quantitation.

### Statistical analysis

Data were analyzed using the generalized linear model of the statistical analysis system (SAS) Software Version 9.2 (SAS Institute Inc., Cary, North Carolina. U.S). The model (5) involved parameters such as PBMC and RNA concentrations as dependent variables, whereas TACs and DTEs were independent variables. The effects of TACs on bovine PBMC and RNA levels were considered significant for p < 0.001. Tukey’s honest significant difference (HSD) test was performed for mean comparisons.

The yield equation for the analyses is given below:

Y_j_ = m +S_j_ + E_j_ (5)

Where:

Y_j_ = Observations of thermal-related performance of PBMCs and Total RNAs.

m = Overall mean

S_i_ = Effect of *j^th^* TACs on Performance of Bovine PBMCs and Total RNAs.

E_j_ = Random error associated with Y_j_ observations for thermal-related performance of bovine PBMCs and Total RNAs.

We further conducted association analyses using Pearson’s correlation analysis of Statistical Package for the Social Sciences Statistics (SPSS) version 20 (IBM, Chicago, IL, USA) to test the relationship between PBMC and total RNA concentrations obtained in this study after TS. The correlation coefficient was considered significant for p < 0.01 at two-tailed.

Finally, we performed regression analysis to predict the reduction of PBMCs and the concentrations of RNA that can be accounted for by heat shock. The regression coefficient was considered significant at p < 0.01.

Both equations of regression and correlation coefficients are as shown below:

i. Linear regression equation

*y* = *a+b(x)*(6)

Where:

*x* = Predictor variable: TACs, an independent variable

*y* = Response variables: PBMCs and RNAs, dependent variables

*a* = TACs intercept which is the plot on the *x*

*b* = Slope of the line

Pearson’s correlation coefficient equation


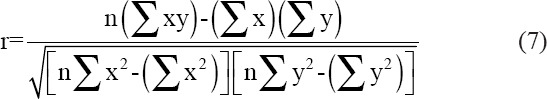


Where:

n = Number of observations

Sx = Total values of PBMCs count

Sy = Total values of RNAs concentration

Sxy = Sum of the product of PBMCs count and RNAs concentration

Sx^2^ = Sum of the squares of PBMCs count

Sy^2^ = Sum of the squares of RNAs concentration.

## Results

### Number and viability of thermally stressed PBMCs

The viability and concentration of PBMCs were measured before and after the TSS procedure. Before TSS, we measured 100% cell viability, indicating that all PBMCs were viable. The concentration of viable PBMCs was estimated to be within the range of 7.04 × 10^6^–2.56 × 10^7^ cells/mL before TSS.

### Effect of different TACs and DTEs on PBMCs and total RNAs

Mean performance values for PBMCs subjected to different TACs and DTEs are summarized in Table-1. Overall, our results showed that harsher TACs and longer DTEs negatively affected the number and viability of PBMCs. For example, the greatest number of PBMCs (2.56 × 10^7^ ± 0.22 cells/mL) and the greatest viability were recorded during the no stress and zero-time thermal duration (0 °C TAC and 0 h-DTE or control). In other words, in the control experiment, PBMCs were not exposed to heat shock and as such they were not affected by prompt apoptosis or cell death. On the other hand, PBMCs that were exposed to extreme TAC (45°C) and 6 h-DTE were the most affected by the heat shock/thermal assault as evidenced by the small cell number (3.59 × 10^5^ ± 0.34 cells/mL) or low cell count after completion of the TSS procedure (Table-1). The other TAC and DTE combinations and their corresponding effects are also shown in Table-1 and followed a similar trend.

**Table-1:** Mean concentration values for PBMCs and total RNAs after thermal stress stimulations at differential TACs and DTEs in Indian Zebu‑Jersey crossbreds.

TAC/DTE	Number of PBMCs (cells/mL)	Concentration of total RNA (ng/μL)
37°C/3 h	1.64×10^6^± 0.12^b^	9.89×10^3^± 0.15^a^
37°C/6 h	9.19×10^5^± 0.11^c^	6.91×10^3^± 0.13^c^
40°C/3 h	7.80×10^5^± 0.22^d^	1.88×10^3^± 0.22^d^
40°C/6 h	4.00×10^5^± 0.33^f^	7.23×10^2^± 0.44^e^
45°C/3 h	6.04×10^5^± 0.45^e^	7.57×10^2^± 0.32^e^
45°C/6 h	3.59×10^5^± 0.34^g^	6.99×10^2^± 0.51^f^
0°C/0 h	2.56×10^7^± 0.22^a^	8.95×10^3^± 0.15^b^

^a, b, c, d, e, f, g^ Means within the same column having different superscripts are significantly different ***p < 0.001, TACs=Thermal assault conditions, DTEs=Durations of thermal exposures, PBMCs=Peripheral blood mononuclear cells

We also determined the concentration of total RNA obtained from PBMCs subjected to TACs. Different TACs and DTEs significantly affected (p < 0.001) the total RNA concentration in stressed PBMCs. The PBMCs that were exposed to the extreme TAC of 45°C and 6 h-DTE had the lowest (p < 0.001) concentration of total RNA (699.50 ± 0.51 ng/μL), whereas PBMCs exposed to a normal 37°C TAC for 3 h-DTE had the highest total RNA concentration (9893.00 ± 0.15 ng/μL), which was very close from the RNA concentration measured in PBMCs exposed to no TAC and zero TDE (8948.00 ± 0.15 ng/μL), as shown in Table-1.

**Figure-4:**
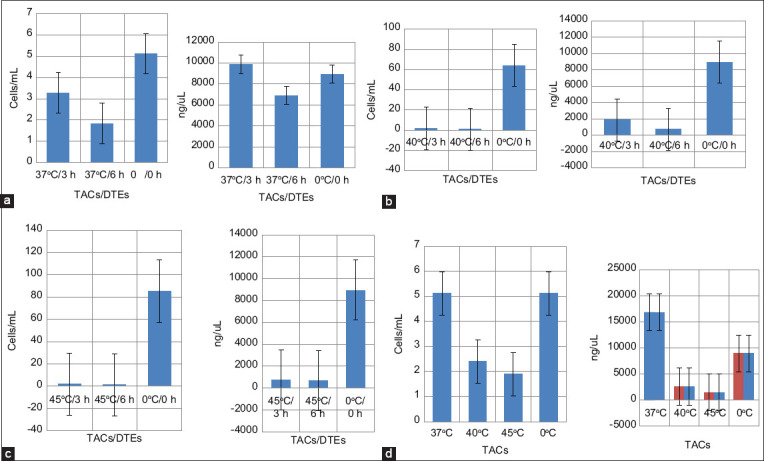
Comparative assessment viability of peripheral blood mononuclear cells and RNA concentrations *in* *vitro* thermal stress stimulation (a) at 37°C; (b) 40°C; and (c) 45°C; (d) under differential thermal assault conditions were significantly different ***p < 0.001.

Figure-4a–c shows a graphical depiction of how the cells of a bovine species respond to TSS with a decrease in the cell number, viability, and RNAs concentration in PBMCs with harsher TACs and longer DTEs. Harsher TACs combined with varying DTEs induced lower PBMC count and viability, and consequently, the total RNA concentration exponentially declined (Figure-4d).

We determined the association between the number of PBMCs and total RNA concentration after completion of the heat shock procedure using Pearson correlation analysis. A very strong positive association (p < 0.01) was detected between these two variables (PBMCs and RNAs). The coefficients of correlation (r) are presented in Table-2 and showed a similar trend both within and between treatments. Furthermore, we found a 99.8% relationship between the number of PBMCs after thermal shock and the total RNA concentration, as shown in Table-2.

**Table-2:** Relationship analyses between performances of PBMC count and concentration of total RNA at different thermal assault conditions.

Thermal assault conditions	Concentration of total RNA (r)	Significant levels
Numbers of PBMCs at 37°C	86.4	**
Numbers of PBMCs at 40°C	89.2	**
Numbers of PBMCs at 45°C	84.5	**
Numbers of PBMCs at 0°C	00.0	NS
Numbers of PBMC counts between TACs	99.8	*

Correlation coefficient is significant at

** p < 0.01 and

* p < 0.05, NS=Not significant, PBMCs=Peripheral blood mononuclear cells

From our results, we predicted the relationship between the predictor variable TACs and response variables PBMCs and RNAs in the thermally shocked samples using a linear regression analysis. We found very high regression coefficients (R) between TACs and the reduced number of PBMCs (85.7%) and a great coefficient of determination R^2^ (73.4%). In addition, TACs were identified as a predictor variable to account for the reduced number of PBMCs and RNA concentration after the heat shock of our samples. All results are summarized in Table-3.

**Table-3:** Prediction of performance of PBMCs count and concentration of RNA under differential thermal assault conditions using regression analysis.

Response variables	Regression coefficient (R)	Coefficient of determination (R^2^)	Significant levels
Regression of PBMCs over TACs	85.7	73.4	**
Regression of RNA over TACs	82.5	68.1	**

Regression coefficient is significant at

** p < 0.01, PBMCs=Peripheral blood mononuclear cells, TACs=Thermal assault conditions

## Discussion

Global warming has grave consequences on livestock production and negative effects on animal production. Thus, there is a need to mitigate the negative effects of climate change on the production and survival of livestock animals. To this aim, it is necessary to understand how animals respond to different TACs and DTEs. Peripheral blood mononuclear cells have been reported as a viable cellular model to study how livestock animals respond to TACs and heat shock [5]. Circulating PBMCs include leukocytes and can be used as a cellular model to gain insight into the response to TS and the mechanisms by which thermal assault causes chronic inflammation and increases the risk of developing many diseases in bovine and a wide range of mammalian species [12–15]. Senescence-related thermal assaults have been reported to induce a loss of the cell’s power to divide and impair cellular functions, including the immunomodulatory activity of immune response cells [16].

Siddiqui *et al*. [6] reported that under extreme TACs, the cellular system suffers a heat shock and as such defense mechanisms become too weak to respond to TS and opportunistic infections. In the present study, we evidenced a direct relationship between harsher *in vitro* TACs and the lower number of PBMCs, whereas no such relationship was found in control samples exposed to no TAC and DTE. This suggests that harsher TACs cause inhibition of cell proliferation, cellular failure, and decline in cellular functions and prompt apoptosis [17]. Heat shock was reported to provoke cell death and a reduction in the numbers of PBMCs [17–19] by inhibiting cell proliferation through a reduction in cellular activities supporting cell proliferation [20, 21]. Costa *et al*. [22] indicated that TACs or heat shocks inhibit cellular proliferation pathways to hinder the distribution of macromolecules required for cell proliferation.

We tested the relationship between the PBMC count and total RNA concentration after heat shock between and within treatment groups. A very strong positive correlation was found between both response variables. This explains why, as the number of PBMCs declined, an equivalent reduction in RNA concentration was recorded. It implies that TACs concurrently impacted the PBMC count and total RNA concentration. We quantified and regressed the number of PBMCs and RNA levels over differential TACs and found regression coefficients (r) of 85.7% for the PBMC count and 82.5% for RNA concentration. This suggests that the increase in differential TACs accounted for an 85.7% decline in the number of PBMCs and an 82.5% reduction in the RNA concentration [23]. However, there was no significant relationship between the quantities of PBMCs and RNA in the control samples, probably because they were not subjected to the *in vitro* TSS procedure.

Furthermore, we calculated the coefficient of determination (R^2^) after thermal assault/heat shock and found R^2^ of 73.4 for the number of PBMCs and 68.1 for the RNA concentration. This implies that for every 100 PBMCs/mL, TACs/heat shock accounts for the death of 73.4 PBMCs and a 68.1 ng/μL reduction of RNA concentration. Our data revealed a regression coefficient (R) greater than the values obtained for the coefficient of determination (R^2^), indicating a very strong relationship between the predictor variable (TACs) and the response variables (PBMC count and RNA concentration) [23]. To the best of our knowledge, no previous comparative study has reported differential responses in the RNA concentration to TACs in Indian Zebu–Jersey crossbreds and/or Indian *Bos indicus* or crossbreds. Thus, there are no previous data to compare our results with.

As evidenced here, PBMCs exposed to the mild/moderate thermal condition of 37°C for 3 h were the least affected by heat shock or thermal assault. This temperature-time combination resulted in the highest PBMC viability, close to that of the control samples (the ones which were not exposed to heat shock). This scenario further suggests that a moderate *in vitro* thermal condition (37°C for 3 h) mimicked the mammalian BT or provided the BT required by mammals for proper cellular functioning and survival [6]. The present study shows that cells performed better under a mild *in vitro* thermal condition of 37°C and further confirms that 37°C is the normal BT required for optimal biological functions of the body.

Some studies have shown that exposure of cells to mild/low temperatures such as 37°C increases cell growth rate, survival, and viability and affects embryo development [24, 25]. This is possible because the normal BT (37°C) provides a suitable physiological environment for Taq polymerase, which has a substantial enzymatic activity for DNA synthesis [26]. Moreover, it was proven that exposure of cells to moderate/mild TACs or heat shock enhances cell survival and growth [8, 27]. The present *in* *vitro* TSS of bovine PBMCs confirmed that cellular systems of Indian Zebu–Jersey crossbred cattle are vulnerable to heat shock and thermal assault. The PBMCs were reduced in number and underwent cell death at higher/extreme temperatures. This implies that exposing animals to extreme sunshine intensity or thermal conditions of tropical environments cause devastating cellular failure or shutdown, leading to a reduced number of cells or low cell count, cellular inhibition, impairment of the optimum performance, and possible cell death or prompt apoptosis [17].

In an *in vitro* study involving bovine and human species, PBMCs were reported to suffer under TACs [21, 28]. It was reported that long-lasting and extreme TACs caused an exponential reduction in the cellular response of lymphocytes to mitogens. During cellular failure occasioned by different TACs and DTEs, lymphocytes were shown to malfunction because thermal assaults cause a decrease in lymphocyte levels. In addition, a thermal assault decreases the viability of PBMCs and increases the expression of some immune response genes, such as toll-like receptors, interleukins, and cytokines, which contribute to the body’s defensive mechanisms against pathogenic infections [26].

The response of immune cells, including lymphocytes, under heat shock to fight foreign bodies is impaired, thereby rendering animals vulnerable to pathogenic infections [13] and leading to poor health conditions, decreased production performance, and economic loss. An earlier *in vitro* study on cell proliferation in native and crossbred dairy cattle and reports derived from a previous *in vivo* TSS investigation on PBMCs of Bama miniature pigs proved that prolonged exposure to severe heat shock is responsible for a decline of the reactivity of immune cells such as lymphocytes. This may contribute to the higher occurrence of some pathogenic infections under harsh thermal environmental conditions [21, 29, 30].

In the present study, we detected a direct proportional influence of TAC-DTE combinations on the total RNA concentration in stressed PBMCs, with RNA concentration decreasing as TACs went from moderate to severe. Previous studies [9, 21] showed that *in vitro* incubation at temperatures simulating hyperthermia subsequently impaired nucleotide synthesis in mitogen-stimulated PBMCs. Moreover, several reports found that RNA as a nucleic acid is heat labile and very unstable and highly denatured under TACs [6]. Consequently, TAC is an environmental factor that can cause mutational damage to the nucleic acid structure, synthesis, and functions.

Our investigations showed that RNA concentrations in PBMCs exposed to moderate TSS (37°C for 3 h-DTE) were the highest, followed by the levels found in the control experiment (0°C for 0 h). In contrast, the lowest RNA concentrations were found in PBMCs exposed to 45°C for 6 h-DTE. This might be due to the fact that a temperature of 37°C *in vitro* mimics the normal BT of mammals and enhances the viability of cells [8]. Siddiqui *et al*. [6] reported that different thermal stimulations of cells can undermine the length of the cell cycle and the synthesis of nucleic acids in the S phase of the cycle as well as degradation of nucleic acid [6]. Furthermore, a long “S” phase indicates a higher potential for cell proliferation which usually happens under moderate thermal conditions at 37°C. In addition, this study reported higher cell viability under a normal TAC of 37°C.

In normal BT (37°C) conditions, there is an elevation of nucleic acid synthesis due to the introduction of extracellular protein kinase and phosphoinositide 3-kinase/Akt signal transduction during transcription [31, 32]. Furthermore, increased cell proliferation, cell viability, and nucleic acid synthesis were shown to happen for longer S and G2/M phases of the cell cycle [7, 9]. A recent report also indicated that cells at the G1/S or G2/M checkpoints and in the S phase are more sensitive to death and arrest of proliferation when exposed to TACs [10].

## Conclusion

It can be concluded that PBMCs of Indian Zebu-Jersey crossbreds were greatly affected by heat shock/thermal assault at 45°C for 6 h-DTE more than at any other thermal conditions studied. Prolonged and extreme TACs such as high tropical temperatures expose the cellular systems of livestock animals to heat shock, thereby preventing optimal cell performance and possibly leading to cell death or prompt apoptosis. Therefore, harsh TAC-DTE combinations negatively affect cell count and viability. They cause prompt necrosis or cell death. Thus, PBMCs can be used as cellular model/indicators to understand the response of animals to thermal assaults *in vitro* and *in vivo*. The association between the reduced PBMC count after *in vitro* TSS and the expression of the heat shock protein 70 gene will be determined in the future to understand further how India Zebu–Jersey crossbreds respond to *in vitro* thermal conditions. This will be used to study *in vivo* response of Indian Zebu-Jersey crossbreds to different environmental TACs and will further enable *in vivo* understanding of the thermotolerance potential of bovine species under life scenarios/conditions for better adaptation, survival, and production performance.

## Authors’ Contributions

GOO: Conceptualized and designed the research work, drafted the manuscript, and performed the experiment. GMM: Revised the manuscript. AKT, NM, RS, AR, and MJ: Carried out the blood sample collection, data collection, management of the experimental animals, and structured the scientific content. MO and COI: Contributed the reagents, summarized the data, and performed the statistical analyses. All authors have read and approved the final manuscript.
